# Local extinction of the Asian tiger mosquito (*Aedes albopictus*) following rat eradication on Palmyra Atoll

**DOI:** 10.1098/rsbl.2017.0743

**Published:** 2018-02-28

**Authors:** Kevin D. Lafferty, John P. McLaughlin, Daniel S. Gruner, Taylor A. Bogar, An Bui, Jasmine N. Childress, Magaly Espinoza, Elizabeth S. Forbes, Cora A. Johnston, Maggie Klope, Ana Miller-ter Kuile, Michelle Lee, Katherine A. Plummer, David A. Weber, Ronald T. Young, Hillary S. Young

**Affiliations:** 1 Western Ecological Research Center, US Geological Survey, Santa Barbara, CA 93106, USA; 2 Marine Science Institute, University of California Santa Barbara, Santa Barbara, CA 93106, USA; 3 Department of Ecology Evolution and Marine Biology, University of California Santa Barbara, Santa Barbara, CA 93106, USA; 4 Department of Entomology, University of Maryland, College Park, MD 20742, USA; 5 Department of Biology, Stanford University, Standford, CA 94305, USA

**Keywords:** secondary extinction, extirpation, *Rattus*, *Culex*

## Abstract

The Asian tiger mosquito, *Aedes albopictus,* appears to have been extirpated from Palmyra Atoll following rat eradication. Anecdotal biting reports, collection records, and regular captures in black-light traps showed the species was present before rat eradication. Since then, there have been no biting reports and no captures over 2 years of extensive trapping (black-light and scent traps). By contrast, the southern house mosquito, *Culex quinquefasciatus,* was abundant before and after rat eradication. We hypothesize that mammals were a substantial and preferred blood meal for *Aedes*, whereas *Culex* feeds mostly on seabirds. Therefore, after rat eradication, humans and seabirds alone could not support positive population growth or maintenance of *Aedes*. This seems to be the first documented accidental secondary extinction of a mosquito. Furthermore, it suggests that preferred host abundance can limit mosquito populations, opening new directions for controlling important disease vectors that depend on introduced species like rats.

## 1. Background

Introduced rats threaten native species (like seabirds), cause economic damage, and can transmit diseases to humans [1]. In response, humans invest billions of dollars in rat control. However, rat eradications have not been linked to mosquito extirpations. This is consistent with the assumption that blood-feeding success does not limit mosquitoes [2]. Under such assumptions, rat declines should increase mosquito bites on humans owing to vector switching (electronic supplementary material). However, because egg laying typically requires a blood meal, it makes sense that mosquito abundance, and perhaps persistence, would depend on blood-feeding success, which itself should increase with host density and suitability [3]. If so, removing preferred hosts such as rats could drive mosquitoes to secondary extinction, thereby reducing bites on humans [4,5] (electronic supplementary material).

**Figure 1.**
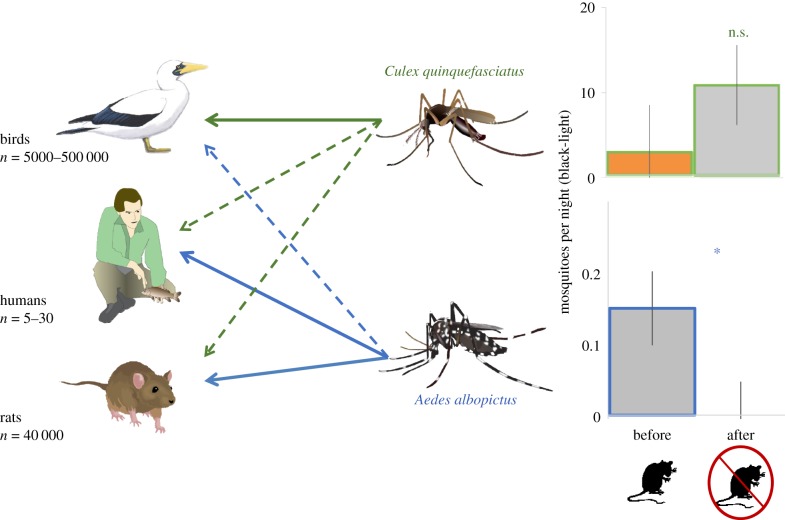
*Aedes*, primarily a mammal feeder, likely lost its main blood meal after rat eradication, leading to its coextinction. In contrast, *Culex* uses seabirds and shorebirds as preferred hosts and was less impacted by rat eradication (bars show means and s.e.). Solid lines indicate primary hosts; dashed lines indicate incidental hosts. (Online version in colour.)

Palmyra Atoll has no native mosquitoes, but its wet tropical climate is suitable for the southern house mosquito, *Culex quinquefasciatus* (hereafter *Culex*), and the Asian tiger mosquito, *Aedes albopictus* (hereafter *Aedes*). *Culex* was introduced during World War II [6]; it is small and bites at night, feeding mostly on birds, but also on mammals, including humans [7] (figure 1, top-left panel). Through its bites, this *Culex* species can vector lymphatic filariasis, West Nile fever and Japanese encephalitis. *Aedes* arrived sometime before 2002, when Chris Depkin collected larvae and adults (Bishop Museum accession no. 2018.003). Adult females are large, aggressive day-time biters, with conspicuous striped coloration, preferring mammals, including rats [7], but some *Aedes* populations will feed on birds when mammals are not available [7] (figure 1, bottom-left panel). This *Aedes* species can vector lymphatic filariasis, yellow fever, Rift Valley fever, dengue fever, chikungunya and Zika [8]. Although there have been no documented vectored diseases on Palmyra, both mosquitoes were a nuisance.

In June 2011, the approximately 40 000 rats on Palmyra were eradicated by applying brodifacoum [9]. After rat eradication, rat prey, like palm seedlings and crabs, increased [10]. Although mosquitoes still harassed people in the evenings, visitors found it unnecessary to apply mosquito repellent during the day and began to suspect *Aedes* had been extirpated.

## 2. Material and methods

Palmyra Atoll National Wildlife Refuge (5°52′ N, 162°04′ W) lacks an indigenous human population. The atoll has a saltwater lagoon encircled by two-dozen natural and created islets covered by introduced coconuts or tall native trees and shrubs. Breeding seabirds nest in the native forests, whereas shorebirds use the flats and shoreline. Although there are no native mammals and few native insects [11], humans (especially a US military occupation in World War II) have introduced rats and many plant and insect species.

In 2009, before rat eradication, we surveyed flying insects with black-light traps (John Hock, New Standard Miniature BlackLight (UV) Trap Model 1212, unbaited, no CO_2_) across 15 islets during 54 nights (electronic supplementary material). All traps were hung 1–2 m above ground level, 1–2 h before dusk and collected 2–3 h after dawn (details in [12]).

After rat eradication, we (i) surveyed researchers about when and how often they were bitten by mosquitoes (see electronic supplementary material), (ii) intensified mosquito survey efforts, and (iii) modified a model [5] describing conditions for mosquito extirpation (electronic supplementary material). We completed 53 trap-nights across 25 islets in 2015 and 80 trap-nights across 24 islets in 2016 (see electronic supplementary material). We also conducted a year-long mosquito-monitoring programme near the main camp on Cooper Island. From 7 December 2015 to 7 August 2017, we hung a black-light trap in the forest, 100 m from camp. This trap was deployed overnight, once a week (during dry weather) 54 times over 20 months. We also used scent traps after rat eradication, because these are more effective than black-light traps for sampling *Aedes* [13]. Scent traps were hung in high human-use areas for eight continuous months (7 January 2015 to 27 June 2015, replacing scent on 22 March 2015). Then, in July and August 2015 we hung a scent trap for 72 h on the three islands where *Aedes* were detected by black-light trap in 2009.

We compared each mosquito species captured per trap-night before and after eradication using a general linear mixed model with island as a random effect (using the square-root transformation on mosquito count to help meet normality assumptions, though we present the untransformed means below). We also calculated the pooled proportional abundance of species trapped (i.e. *Aedes*/(*Culex* + *Aedes*)), allowing us to estimate percentage of *Aedes* (± 95% CI, binomial exact method) before and after rat eradication.

## 3. Results

**Figure 2.**
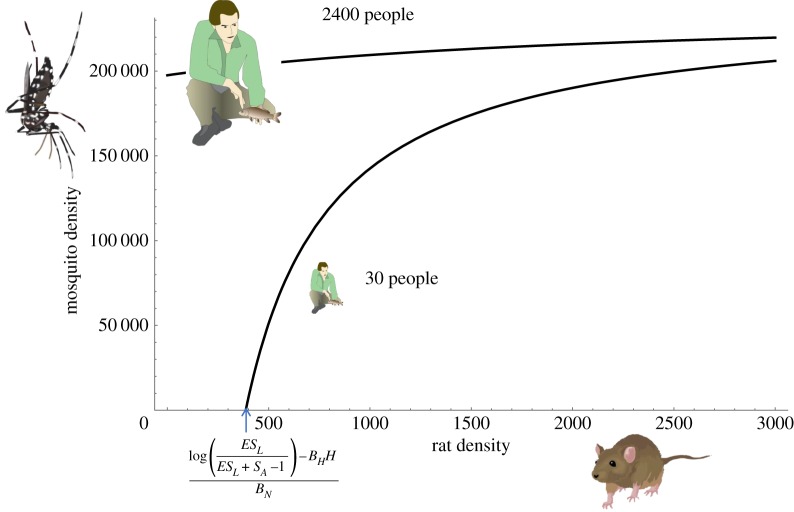
Modelled relationship between *Aedes* density and host densities predicts that *Aedes* should not persist without rats and with few humans (electronic supplementary material). (Online version in colour.)

In 2009, before rat eradication, *Aedes* (0.03 ± 0.40 s.e. per trap set) were present, but less abundant per black-light trap-night than were *Culex* (2.46 ± 0.40 s.e.) (*p* < 0.0001, figure 1, right panel), though, because *Aedes* are less likely to be captured by black-light traps [13], this does not imply that *Aedes* were less abundant than *Culex*. Pooling counts per mosquito species across traps suggested that *Aedes* composed 5.9% (2.6%–11.3%, 95% CI) of the mosquito individuals in black-light traps before rat eradication. A simple model suggests that *Aedes* could persist on a dense rat population (e.g. the approximately 40 000 rats present in 2009) or a dense human population (e.g. the 2400 military personnel in World War II), but not under current conditions with no rats and 5–30 humans (figure 2).

After rat eradication, researchers were bitten less often, and almost always at night (electronic supplementary material). The increased sampling effort captured 35-fold more mosquitoes than before rat eradication (electronic supplementary material). There was no significant difference in *Culex* caught per trap-night before (2.7 ± 5.5 s.e.) than after (10.6 ± 4.7 s.e.) rat eradication (*p* = 0.69). However, the zero *Aedes* caught per black-light trap-night after (0 ± 0.047 s.e.) was significantly less than before (0.15 ± 0.052 s.e.) rat eradication (*p* = 0.0004, figure 1, bottom-right panel). In the pooled samples, *Aedes* composed none (0.00%–0.22%, 95% CI) of the mosquitoes in black-light traps. Pooling the more sensitive scent trap data gave us more confidence that *Aedes* composed none (0.00%–0.12%, 95% CI) of the mosquitoes after rat eradication.

In summary, *Culex* persisted after rat eradication, while *Aedes* went from being present even in non-targeted trapping efforts before rat eradication to undetectable after rat eradication, despite much greater, and more targeted trapping effort (table 1).

**Table 1.** Relative mosquito abundance, by species, before (2009) and after (2015–2016) rat eradication (2011) using black-light traps, scent traps and biting observations. n.a., not available.

species	method	sensitivity	rats	no. rats
*Culex*	black-light	moderate	common	common
*Culex*	scent	moderate	n.a.	common
*Culex*	anecdotal	highest	common	common
*Aedes*	black-light	low	uncommon	absent
*Aedes*	scent	high	n.a.	absent
*Aedes*	anecdotal	highest	common	absent

## 4. Discussion

Had rat densities simply been reduced rather than eradicated, or had human densities been higher after rat eradication, biting rates on humans could have increased as mosquitoes switched from rats to humans (see figure in electronic supplementary material). Instead, bites from *Aedes* ceased. Our inability to document an *Aedes* bite, or trap an *Aedes* mosquito, over 2 years of sensitive surveillance meets the World Health Organization's standards for demonstrating mosquito eradication [8]. Most mosquito eradications are fleeting, because mosquitoes can soon recolonize. For instance, cycles of *Aedes* eradication and reintroduction followed intensive spraying on Kwajalein Island [14]. The Palmyra eradication seems different: lack of recovery over 6 years suggests that conditions on Palmyra have become unsuitable for *Aedes*.

We hypothesize that *Aedes* was eradicated from Palmyra primarily because its persistence depended on taking blood meals from rats (figure 1). Rat eradication could have also reduced larval habitat because rats open coconut husks, creating suitable habitat for container-breeders like *Aedes* and *Culex* [15]. Larval habitat might have also declined after the 2011 rat eradication, because 2011 and 2012 were drier than average years. However, rainfall records since 2002 indicate both wetter and dryer than average years before and after rat eradication, with no prolonged droughts (see electronic supplementary material), suggesting that such dry periods would not have eradicated *Aedes* on their own. Therefore, we expect this *Aedes* eradication will last as long as rats fail to re-invade Palmyra.

Although there are few documented coextinctions [16], examples include 10 parasitic trematode species that went locally extinct after their snail host was extirpated [17], a rat tick that went extinct along with the Christmas Island rat [18], and 11 bird lice species extirpated when their island-endemic bird hosts went extinct [19]. In fact, *Aedes* is not the only putative secondary extinction associated with the Palmyra rat eradication. Most rats on Palmyra were parasitized with a rodent-specific stomach nematode [20], which must have also gone extinct on the atoll after rat eradication. These changes in the Palmyra food web show how removing introduced rats can have unintended indirect effects, including eradicating an introduced disease vector.

## Supplementary Material

Modeling mosquito abundance as a function of two host densities

## Supplementary Material

Sampling locations, types, and effort for mosquitoes before and after rat eradication

## Supplementary Material

Researcher survey methods and results

## Supplementary Material

Mosquito trap results from Palmyra Atoll, by date, island and trap type

## Supplementary Material

Annual rainfall on Palmyra Atoll taken from the station rain gauge

## Data accessibility

Datasets have been uploaded as electronic supplementary material.
