# Supplementary material for: Local extinction of the Asian tiger mosquito (Aedes albopictus) following rat eradication on Palmyra Atoll
Source: Biol Lett. 2018 Feb 28;14(2):20170743. doi: 10.1098/rsbl.2017.0743 (PMC5830668; doi:10.1098/rsbl.2017.0743)
Supplement: Modeling mosquito abundance as a function of two host densities [file rsbl20170743supp1.docx]

Modeling mosquito abundance as a function of two host densities

Electronic supplementary material for: Local extinction of the Asian tiger mosquito (*Aedes albopictus*) following rat eradication on Palmyra Atoll

Kevin D. Lafferty, John P. McLaughlin, Daniel S. Gruner, Taylor A. Bogar, An Bui, Jasmine N. Childress, Magaly Espinoza, Elizabeth S. Forbes, Cora A. Johnston, Maggie Klope, Ana Miller-ter Kuile, Michelle Lee, Katherine A. Plummer, David A. Weber, Ronald T. Young, Hillary S. Young

Modeling can identify the critical host suitability and abundance below which mosquito populations go extinct. Although most malaria transmission models assume adult mosquitoes have access to unlimited blood meals, some models consider that mosquitoes must feed to survive [1,2]. Sota and Mogi [2] modeled how increasing a wildlife or domestic animal reservoir could affect biting rates (and thereby disease transmission) on humans. Bites on humans are a product of mosquito density, biting rates per mosquito, and the proportion of bites directed at humans. Increasing a non-human reservoir population (like rats) can increase or decrease biting rates on humans, depending on what determines mosquito carrying capacity. Most models assume that larval habitat sets carrying capacity. Their model assumes that mosquitoes need both larval habitat and blood meals to complete their life cycle. The more limited larval habitat is, the more increasing a reservoir population decreases biting rates on humans. This is because increasing the reservoir does not increase the habitat-constrained mosquito population much, but it increases the ratio of reservoirs to humans available for a mosquito to bite, thereby decreasing the proportion of bites directed to humans. This phenomenon is termed zooprophylaxis when referring to domestic reservoirs, and the dilution effect when referring to wildlife reservoirs. However, when blood meals limit mosquito abundance more than does larval habitat, increasing a reservoir population increases the mosquito population, and this can lead to increased biting rates on humans. It is easy to imagine a hump-shaped relationship between reservoir host density and biting rates on humans. In the extreme case, the mosquito can be extirpated if the reservoir population is below a critical threshold, eliminating bites on humans.

Sota and Mogi’s [2] model has a mosquito population with larval (*S_L_*) and adult (*S_A_*) survival rates, that bite humans (*H*) at a per capita rate *B*_H_ and other animals (*N*) at per capita rate *B*_N_, Egg laying *E*, and density dependence δ. In figure 2 (main text), we consider Sota and Mogi’s [2] mosquito density, *M*, as a function of animal density, using their (relabeled) equation (7), and their parameters: *S_A_* = 0.6, *S_L_*= 0.1, *B_H_* = 0.002, *B*_N_ = 0.0002, *E* = 50, *δ* = 0.000001, with the exception that we reduced *B_H_* from 0.002 to 0.0002, as this yielded a daily bite rate on humans ~ 5, which seemed more reasonable from our experience than the alternative ~ 50.

$$M=\frac{-1+S_{A}+\left( 1-ⅇ^{-B_{H}H-B_{N}N} \right)E S_{L}}{\left( 1-ⅇ^{-B_{H}H-B_{N}N} \right)E\left( 1-S_{A} \right)\delta}$$

We plot *M* for two human population sizes *H* relevant for Palmyra Atoll: 30 people during the research season, and 2400 people during WWII. Mosquitoes go extinct (*M* = 0), when non-human animal density (*N*) is below:

$$\frac{\mathrm{Log}[\frac{ES_{L}}{ES_{L}+S_{A}-1}]-B_{H}H}{B_{N}}$$

We note that these parameters are not confirmed for Palmyra Atoll, so are not meant to imply an empirical outcome. Rather, they are used to show that a simple model suggests that mosquitoes can be driven extinct if their non-human host becomes rare. This solution illustrates some key points. First, if a suitable human population (i.e., *Aedes’* most suitable host) is sufficiently abundant ($H>\mathrm{Log}\left[ \frac{ES_{L}}{ES_{L}+S_{A}-1} \right]/B_{H}$), the mosquito will persist with or without additional, less suitable, animal hosts. Second, at lower human densities, a minimum product of animal host density and suitability emerges below which the mosquito population will not persist. Third, reducing larval survival (e.g., by reducing breeding habitat abundance or quality, for instance rats no longer open coconut husks on Palmyra Atoll) makes the mosquito population even more sensitive to losing a suitable animal host. These results help to explain how the *Aedes* population could have been extirpated, despite the presence of potential blood meals from humans and birds. One could explore this model further by estimating relative biting rates from blood meal analysis (e.g., humans, birds, rats) and making empirical predictions about secondary extinctions. Furthermore, it implies that simply reducing rat populations could increase bite rates from *Aedes* on humans, thereby increasing transmission for several important human diseases, whereas extirpating mosquitoes reduces bite rates to 0 (Figure 1 ESM).

Mosquito bites per person,

per day


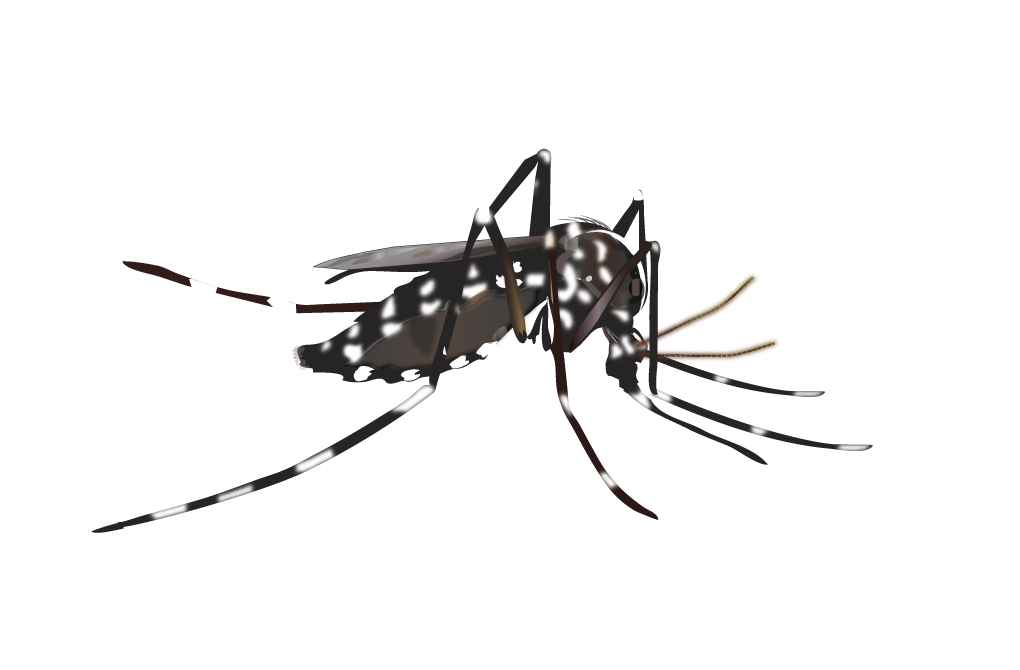


Rat

density


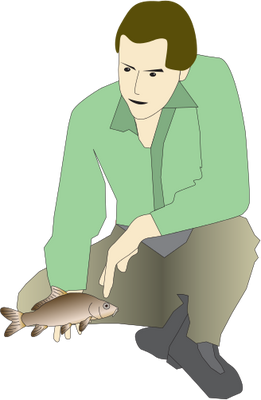


30 people

2400 people


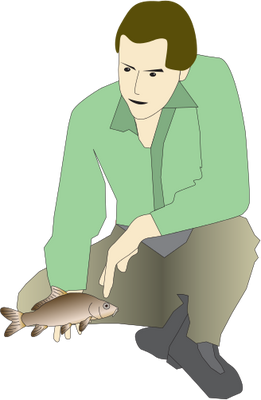

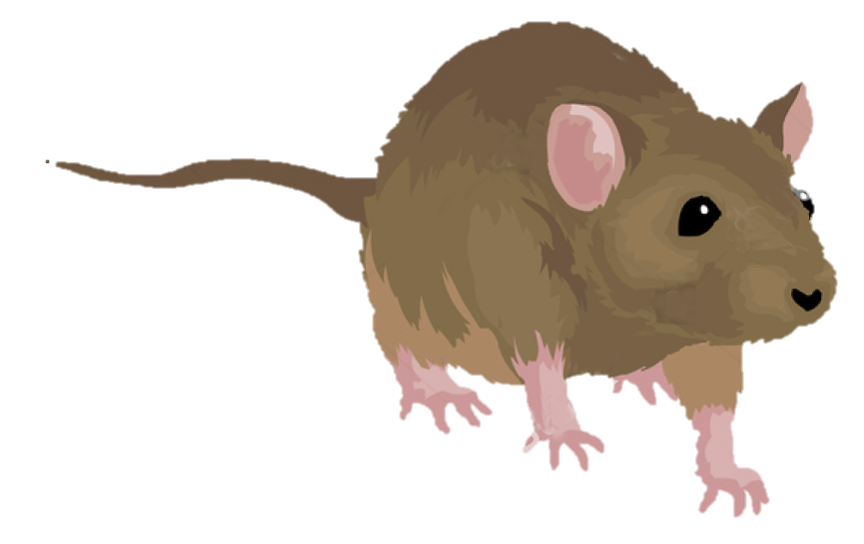


Figure 1. ESM. Bites per person as a function of rat density for two human densities. Increased rat density diverts bites away from people. But when humans and rats are at low densities, bites per person declines as a lack of hosts causes mosquito density to decline.

We hypothesize that the generalist *Culex* has persisted on Palmyra by feeding on its preferred hosts, the seabirds and shorebirds that are abundant on the Atoll. In contrast, female *Aedes*, as mammal specialists, have lost a critical blood source. Humans were the only source of mammal blood on Palmyra after rat eradication. During peak research seasons, up to 30 humans lived on Palmyra. But during the winter seasons, there were as few as four people on the Atoll. Even though humans are large compared to rats, the blood in four people (about 20 L) might not have been sufficient to maintain the *Aedes* population, especially in comparison the estimated 40,000 rats that populated Palmyra before eradication [3]. At 25 ml blood per rat, this translates to about 1000 L rat blood for *Aedes* to feed on. With rats eradicated, the main option for blood meals is birds. Birds can supply some *Aedes’* blood meals (17% in Missouri [4], 7% in North Carolina [5], 6% in Hawaii [6], 0% in Baltimore (LaDeau et al. in review). However, the *Aedes* on Palmyra have not persisted on bird blood alone.

**References**

[1] Aron JL, May RM. 1982 The population dynamics of malaria. In *The population dynamics of infectious diseases: theory and applications* 139-179. New York, NY: Springer. (doi:10.1007/978-1-4899-2901-3_5)

[2] Sota T, Mogi M. 1989 Effectiveness of zooprophylaxis in malaria control: a theoretical inquiry, with a model for mosquito populations with two bloodmeal hosts. *Med. Vet. Entomol.* **3**, 337-345. (doi:10.1111/j.1365-2915.1989.tb00240.x)

[3] Wegmann A, Middleton S. 2008 Trip report, Jarvis Island National Wildlife Refuge and Palmyra Atoll National Wildlife Refuge visit and terrestrial assessment, 20 March to 8 April 2008. *US Fish and Wildlife Service-PRINWRC*.

[4] Savage H, Niebylski M, Smith G, Mitchell C, Craig Jr G. 1993 Host-feeding patterns of *Aedes albopictus* (Diptera: Culicidae) at a temperate North American site. *J. Med. Entomol.* **30**, 27-34. (<https://doi-org.proxy.library.ucsb.edu:9443/10.1093/jmedent/30.1.27>)

[5] Richards SL, Ponnusamy L, Unnasch TR, Hassan HK, Apperson CS. 2006 Host-feeding patterns of *Aedes albopictus* (Diptera: Culicidae) in relation to availability of human and domestic animals in suburban landscapes of central North Carolina. *J. Med. Entomol.* **43**, 543-551. ([https://doi-org.proxy.library.ucsb.edu:9443/10.1603/0022-2585(2006)43[543:HPOAAD]2.0.CO;2](https://doi-org.proxy.library.ucsb.edu:9443/10.1603/0022-2585(2006)43%5b543:HPOAAD%5d2.0.CO;2))

[6] Tempelis C, Hayes R, Hess A, Reeves W. 1970 Blood-feeding habits of four species of mosquito found in Hawaii. *Am. J. Trop. Med. Hyg.* **19**, 335-341. (https://doi-org.proxy.library.ucsb.edu:9443/10.4269/ajtmh.1970.19.335)
