# Supplementary material for: Local extinction of the Asian tiger mosquito (Aedes albopictus) following rat eradication on Palmyra Atoll
Source: Biol Lett. 2018 Feb 28;14(2):20170743. doi: 10.1098/rsbl.2017.0743 (PMC5830668; doi:10.1098/rsbl.2017.0743)
Supplement: Sampling locations, types, and effort for mosquitoes before and after rat eradication [file rsbl20170743supp2.docx]

Sampling locations, types, and effort for mosquitoes before and after rat eradication

Electronic supplementary material for: Local extinction of the Asian tiger mosquito (*Aedes albopictus*) following rat eradication on Palmyra Atoll

Kevin D. Lafferty, John P. McLaughlin, Daniel S. Gruner, Taylor A. Bogar, An Bui, Jasmine N. Childress, Magaly Espinoza, Elizabeth S. Forbes, Cora A. Johnston, Maggie Klope, Ana Miller-ter Kuile, Michelle Lee, Katherine A. Plummer, David A. Weber, Ronald T. Young, Hillary S. Young
