# Supplementary material for: Local extinction of the Asian tiger mosquito (Aedes albopictus) following rat eradication on Palmyra Atoll
Source: Biol Lett. 2018 Feb 28;14(2):20170743. doi: 10.1098/rsbl.2017.0743 (PMC5830668; doi:10.1098/rsbl.2017.0743)
Supplement: Researcher survey methods and results [file rsbl20170743supp3.docx]

Researcher survey methods and results

Electronic supplementary material for: Local extinction of the Asian tiger mosquito (*Aedes albopictus*) following rat eradication on Palmyra Atoll

Kevin D. Lafferty, John P. McLaughlin, Daniel S. Gruner, Taylor A. Bogar, An Bui, Jasmine N. Childress, Magaly Espinoza, Elizabeth S. Forbes, Cora A. Johnston, Maggie Klope, Ana Miller-ter Kuile, Michelle Lee, Katherine A. Plummer, David A. Weber, Ronald T. Young, Hillary S. Young

To quantify the change in mosquito biting behavior and frequency we surveyed researchers and staff who spent >2 weeks on island with an email survey. Survey recipients were asked to answer all the following questions about their experiences on Palmyra atoll.  Recollections from 2011 (the year of eradication) were not included in the survey as it was not clear how soon mosquitos would have disappeared from the atoll.

**1. Did you notice mosquitoes (in the air, while working, etc.) during your time on Palmyra?**

**2. Did you get mosquito bites during your time on Palmyra?**

**3. Were mosquitos 1) common 2) rare or 3) absent *during the day* during your time on Palmyra?**

**4. Were mosquitos 1) common 2) rare or 3) absent *during the evening/dawn/or night*during your time on Palmyra?**

**5. Did you use mosquito repellant during your time on Palmyra? if so please indicate if you applied the repellant durin5g day or night.**


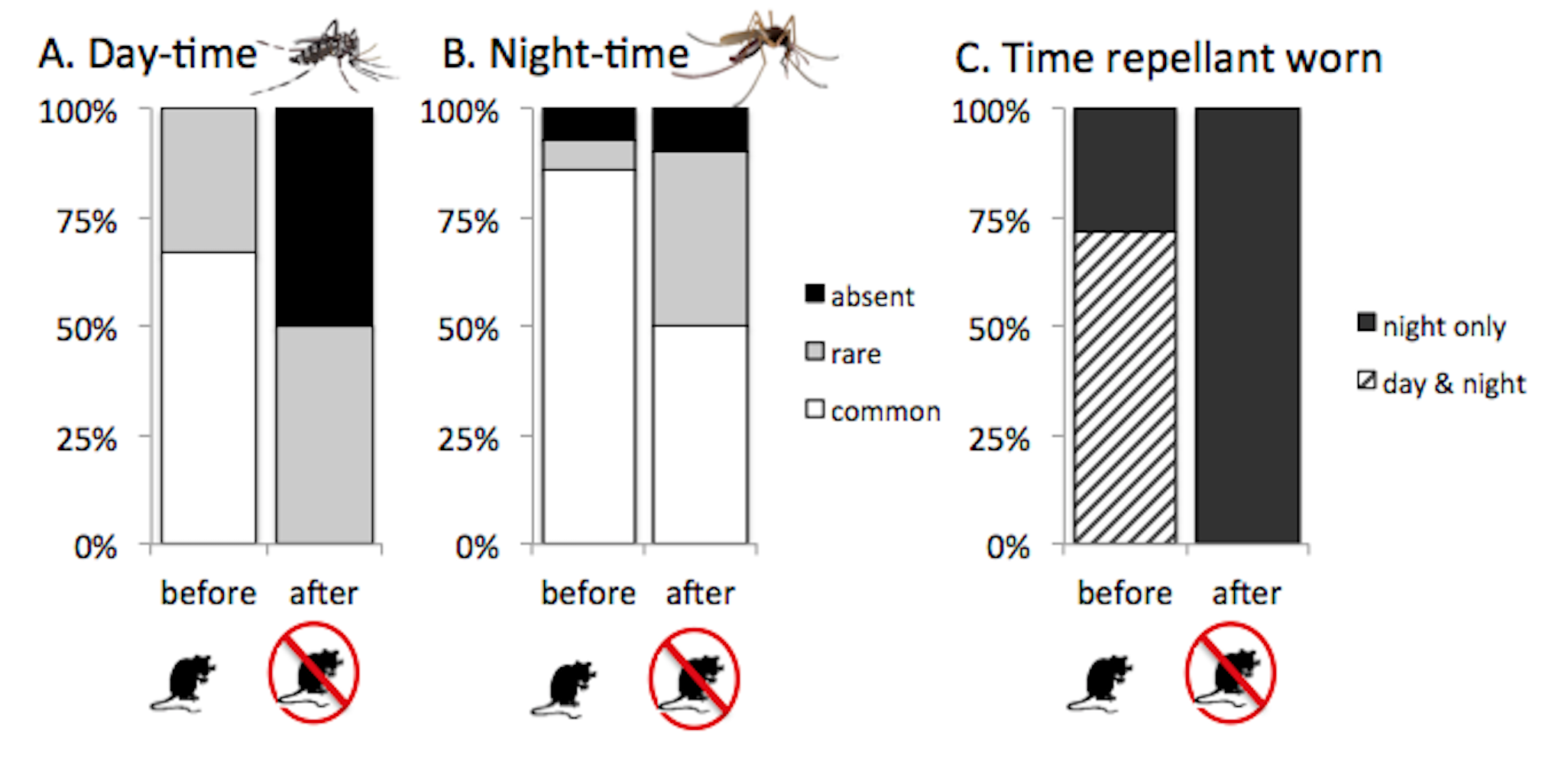


Figure 1. Responses of survey participants (n = 18 pre-eradication, n = 21 post eradication) to questions about A) frequency of mosquitoes observed at day, B) frequency of mosquitoes observed at night, and C) time of day mosquito repellant worn.
